# Regulation of the veterinary profession: the authorization to practice, origins, milestones, and emerging challenges

**DOI:** 10.3389/fvets.2026.1813738

**Published:** 2026-06-04

**Authors:** Jan Vaarten, Nancy De Briyne, Joost van Herten, Sarne De Vliegher

**Affiliations:** 1 Veterinary Faculty, Ghent University, Merelbeke-Melle, Belgium; 2 Federation of Veterinarians of Europe, Brussels, Belgium; 3 Royal Veterinary Association of the Netherlands, Houten, Netherlands; 4 Belgium Order of Veterinarians, Merelbeke-Melle, Belgium

**Keywords:** animal welfare, free market, quality, regulated profession, veterinary medicine

## Abstract

Veterinary medicine has evolved over centuries into a profession concerned not only with animal health, but also with public health, food safety, and the wellbeing of ecosystems. Its impact therefore extends beyond individual animals and their owners to society as a whole. To safeguard quality and prevent substandard care, the veterinary profession is subject to regulation. Only suitably qualified individuals are permitted to practice, with authorization systems administered either directly by governments or through professional regulatory bodies. This paper examines the development of regulation as a mechanism for maintaining and enforcing quality in veterinary services across European countries. It explores how “quality” in veterinary medicine is conceptualized and defined, and how regulatory frameworks are implemented in practice. It also highlights significant transformations in the field over recent decades. In light of these developments, the paper evaluates the extent to which regulatory bodies remain capable of fulfilling their mandates and sustaining public trust in the profession. Other regulated domains are considered only where they fall within the authority of the regulatory body—for example, when rules on animal welfare or food safety are incorporated into the profession's Code of Conduct.” The paper argues that, although regulatory frameworks are designed to ensure quality, they are increasingly challenged by new developments such as changing market dynamics, corporatization, and technological change. These pressures raise important questions about their continuing effectiveness. The authors conclude that further research is needed, particularly to assess the implementation and effectiveness of professional regulation, which may lead to recommendations that could improve both the quality of veterinary services and the standing of the profession itself.

## 1 Introduction

Humans have co-evolved with and consistently existed alongside non-human animals. The gradual process of animal domestication marked a significant transition in this relationship, accompanied from its earliest stages by practices aimed at caring for sick and injured animals. Initially, such practices were largely grounded in tradition, spiritual beliefs, and empirical observations (1). Over time, veterinary medicine developed into a scientific and evidence-based discipline, primarily concerned with the diagnosis, treatment, and prevention of animal diseases.

Subsequently, the scope of veterinary medicine expanded beyond individual animal health to encompass broader public health concerns. Issues such as the safety of food of animal origin and the transmission of zoonotic diseases became integral components of the profession (2). This evolution reflects an increasing recognition of the interconnectedness between human, animal, and environmental health.

At the same time, awareness has grown regarding the necessity of ensuring and safeguarding the quality of veterinary services. Given that non-experts often lack the capacity to adequately assess such quality, regulatory intervention has become essential. Authorization systems have therefore been established to ensure that only individuals who meet predefined qualifications are permitted to practice veterinary medicine, thereby preventing substandard or unqualified care.

Beyond formal qualification requirements, maintaining societal trust in the veterinary profession also requires addressing factors that may undermine its integrity. One such factor is the potential erosion of professional autonomy, which may hinder veterinarians' ability to uphold appropriate standards of care. For example, challenges may arise from the limited availability of authorized veterinary medicinal products in certain markets. Such a situation can place veterinarians in ethically complex circumstances where they must balance regulatory compliance with the necessity to prevent animal suffering. Additionally, veterinarians may sometimes face conflicting roles, such as simultaneously acting as advisors to animal owners and as regulatory gatekeepers.

Many existing regulatory frameworks and the institutions underpinning them were established several decades ago. Since then, significant transformations have occurred in both veterinary practice and societal expectations. These include market deregulation, evolving societal attitudes toward animals and their welfare (3), and changes in the organization and management of veterinary practices. Moreover, there are rapid advancements in veterinary science and technology (4, 5), including the increasing role of automation and artificial intelligence (6).

This paper presents a narrative policy review based on legal texts, institutional reports, and selected peer-reviewed literature relevant to veterinary professional regulation. It aims to explore the origins and objectives of the regulation, with a primary focus on the European Union (EU) and its Member States, while also incorporating selected comparisons with other jurisdictions. Furthermore, it explores key developments within and surrounding veterinary practice that may influence both the quality of veterinary care and its societal evaluation. Ultimately, this paper seeks to provide a foundation for future research into how current frameworks for the regulation of the veterinary profession can remain adequate in addressing the evolving needs of both society and the veterinary profession.

## 2 Development of veterinary medicine and the veterinary profession

### 2.1 Brief history

The treatment of diseased animals was historically grounded in tradition, religious beliefs, and empirical practices. It was not until the Age of Enlightenment that veterinary medicine began to develop a more systematic and scientific foundation. In Europe, the first veterinary school was established in 1762 in Lyon. The frequent occurrence of epizootic diseases, such as rinderpest, which caused substantial economic losses and widespread poverty (7), prompted the French monarchy to invest in an institution dedicated to the study of animal diseases, their treatment, and the formal education of practitioners.

From its inception, veterinary education extended beyond the narrow confines of disease control to engage with broader societal concerns, including the rural economy, the strategic deployment of military horses (8), and issues of food security (9). Although the prevention and management of animal disease constituted its principal motivation, early evidence indicates that other societal considerations, like animal welfare, also existed. This is exemplified by correspondence addressed to the institution shortly after its establishment by the French philosopher François-Marie Arouet (Voltaire) and in which he advocated for an improved treatment of animals (10).

In subsequent decades, additional veterinary teaching institutions were established across France and other European countries (11). From the mid-nineteenth century onwards, growing attention was given toward food safety, particularly concerning the risks associated with residues of medicinal products in animal-derived foods. At the same time, advances in microbiology and parasitology enabled a more comprehensive understanding of foodborne hazards. Consequently, traditional meat inspection practices were progressively replaced by approaches grounded in scientific principles. As a result, the role of veterinary medicine—and of well-trained veterinarians—expanded beyond animal health to encompass public health more broadly (12). The societal importance of veterinary medicine is increasingly recognized.

### 2.2 Differentiation qualified veterinarians and empiricists

Across several countries, measures to delineate the boundary between formally qualified veterinarians and other providers of animal health services were progressively implemented. France was among the earliest to institutionalize this distinction: as early as 1766, graduates of veterinary schools were awarded official certificates. In many instances, such initiatives originated within the professional community itself, as academically trained veterinarians sought to differentiate their expertise from that of empirically trained practitioners. Subsequently, as governments increasingly acknowledged the public significance of properly regulated veterinary practice, these professional efforts were reinforced through legislative action.

Belgium represents a pioneering case in this regard. In 1850, it became the first country in which the national government—acting upon the advice of the veterinary profession—enacted legislation governing both the education and the practice of veterinary medicine (13). Comparable developments occurred elsewhere, albeit with varying timelines and scopes. In the United Kingdom, the Veterinary Surgeons Act of 1881 prohibited unqualified individuals from using the professional title of “veterinary surgeon” (14). However, it was not until the Veterinary Surgeons Act of 1948 that the practice of veterinary surgery itself was legally restricted to qualified practitioners (15).

France followed a similar trajectory. Although veterinarians were granted exclusive authority over the control of contagious animal diseases as early as 1881, comprehensive legal protection of the profession was only achieved with the 1938 legislation, which reserved all aspects of veterinary medicine to licensed practitioners. In the Netherlands, the 1874 law regulating the practice of veterinary medicine likewise codified professional exclusivity, stipulating that the exercise of veterinary medicine was permissible only for those who had successfully passed a national examination and obtained a certificate of competence (16).

### 2.3 What falls within the scope of veterinary medicine?

As national authorities increasingly turned their attention to the quality and regulation of veterinary medicine, the need emerged to clarify both the substantive scope of veterinary practice and the categories of individuals permitted to engage in it, as well as the conditions under which such practice is authorized.

In most jurisdictions, rather than adopting a comprehensive and abstract definition of “the practice of veterinary medicine,” legislators have opted for an enumerative approach. They specify those activities that need the involvement of a licensed veterinarian or a veterinary para-professional. This approach is illustrated by the Belgian Law of 28 August 1991 on the practice of veterinary medicine, which provides that “the practice of veterinary medicine consists of performing one or more veterinary acts,” and proceeds to define such acts through a list (17).

At the international level, the World Organisation for Animal Health's Terrestrial Animal Health Code does not formally define “veterinary medicine,” but instead defines a “veterinarian” as a person with appropriate education and registration or licensure by a Veterinary Statutory Body. This reflects a broader emphasis on professional qualifications and legal recognition.

A similar lack of harmonized definitions exists within the European Union, where Member States maintain their own legal frameworks. EU legislation therefore relies on national definitions when needed. For example, Regulation (EU) 2016/429 defines an “official veterinarian” as one authorized by a Member State's competent authority and properly qualified to perform official tasks (see Table 1) (18).

**Table 1:** Comparative overview of definitions of activities reserved for duly licensed veterinarians, as established in national legislation and by the Federation of Veterinarians of Europe.

Examination	
NL	Examining an animal for the purpose of making a diagnosis with a view to preventing, curing, alleviating, recognizing or eliminating a condition, animal disease, zoonosis, disease symptom, defect, or internal or external injury or pain Examining an animal for the purpose of making a diagnosis
BE	Examining the animal's state of health with a view to making a diagnosis and, where appropriate, issuing a certificate in this regard Detecting diseases in animals Examining an animal for the purpose of making a diagnosis
UK	The diagnosis of diseases in, and injuries to, animals including tests performed on animals for diagnostic purposes and the giving of advice based upon such diagnosis
FR	Any act intended to determine the physiological state of an animal or group of animals or its state of health, to diagnose a disease, including behavioral disease, an injury, pain, a malformation, to prevent or treat them, to prescribe medication or to administer them parenterally; - “animal surgery”: any act affecting the physical integrity of the animal for therapeutic or zootechnical purposes
FVE	All material or intellectual interventions that have as their objective to diagnose, treat, or prevent mental or physical disease, injury, pain, or defect in an animal, or to determine the health and welfare status of an animal or group of animals, particularly its physiological status
Treatment and interventions	
NL	Performing physical interventions
BE	Surgical and dental interventions
UK	The medical or surgical treatment of animals The performance of surgical operations on animals
FR	Animal surgery: any act affecting the physical integrity of the animal for therapeutic or zootechnical purposes.
FVE	All veterinary interventions, including food or feed chain activities, affecting public health
Prescription	
NL	Prescribing veterinary medicine or animal feed with a medicinal effect Prescribing or administering an anesthetic or stunning
BE	Prescribing veterinary medicine or animal feed with a medicinal effect
UK	
FR	
FVE	Prescribing veterinary medicine or animal feed with a medicinal effect
Reproduction	
NL	Providing assistance with regard to the birth or removal of a fetus Making an animal sterile Retrieving and transferring embryos or ova
BE	Embryo transplantation in animals
UK	
FR	
FVE	
Foodsafety	
NL	
BE	Ante-mortem and post-mortem examination of animals with a view to determining their suitability for human consumption and with a view to obtaining information on the state of health of the herds of origin
UK	
FR	
FVE	
Various	
NL	
BE	Euthanasia of animals Necropsy of animals
UK	
FR	
FVE	Veterinary certification

NL, The Netherlands. Animals Act of 19 May 2011 Article 1.1.

BE, Belgium. Law of 28 August 1991 on the practice of veterinary medicine.

UK, United Kingdom. Veterinary Surgeons Act 1966, [in Section 27(1)].

FR, France. Rural and Maritime Fishing Code 2010.

FVE, Federation of Veterinarians of Europe. Definition Veterinary Act 2019.

At the professional level, the Federation of Veterinarians of Europe has contributed to conceptual clarification by compiling an overview of veterinary activities that require the intervention of a licensed veterinarian (20). Moreover, the FVE has proposed a normative definition of a “veterinarian” as a professional possessing comprehensive scientific education and legal licensure, who is empowered to carry out all aspects of veterinary medicine in an independent, ethical, and personally accountable manner, with due regard for animal health and welfare, client interests, and societal needs (21).

While substantial similarities and overlaps can be observed among the defined activities, notable differences persist with regard to both their scope and level of detail. For instance, food safety is explicitly included within Belgian legislation, whereas it is absent from the other legal frameworks considered here. Conversely, the prescription of veterinary medicinal products is not explicitly addressed in the United Kingdom's list of veterinary acts. In the Netherlands, an additional qualification is introduced: veterinary activities are only considered as such when performed in a professional context, as part of an occupation to earn a living (22).

Furthermore, in contrast to the national legislations outlined above, the Federation of Veterinarians of Europe explicitly incorporates animal welfare into its definition. It includes “all […] interventions that have as their objective […] to determine the health and welfare status of an animal or group of animals.” However, it remains open to interpretation whether this formulation is intended to imply that the assessment of animal welfare status should be regarded as an activity exclusively reserved for veterinarians.

## 3 What entails quality in veterinary medicine and why is it so important?

### 3.1 Liberal profession

For maintaining the quality of veterinary medicine, it is essential to recognize that veterinary medicine belongs to the category of the liberal professions. This concept can be traced back more than two millennia to Marcus Tullius Cicero (106–43 BC), who distinguished between occupations concerned primarily with the production of material goods and those requiring intellectual judgement and serving a broader societal purpose. As he observed, certain professions demand a higher degree of prudence and yield significant benefit to society, citing examples such as medicine and architecture (80). According to Cicero, such professions, characterized by the need for independent reasoning and practical wisdom, were to be exercised by free and autonomous citizens [cf. (23, 24)].

In contemporary discourse, multiple definitions of liberal professions coexist, as reflected in sources such as the European Court of Justice and Directive 2005/36/EC (25, 26). Despite variations, these definitions consistently emphasize several core characteristics, such as the predominantly intellectual nature of the work, and the requirement of advanced qualifications. Others are the independent and personal delivery of services, and a relationship of trust between professional and client. A core value of liberal professions is the dual responsibility to both individual clients and the public good, underscoring the need for veterinarians to be able to exercise autonomous professional judgement.

The term “profession” itself derives from the Latin *profiteor*, meaning to declare publicly or to commit oneself to a set of shared ethical principles. This notion of public commitment is reflected in professional oaths, such as the veterinary oath in the Netherlands, as well as comparable declarations in other jurisdictions, such as the French *Serment de Bourgelat* and the United Kingdom's declaration upon admission to the profession (27, 28) (Declaration on admission to the profession). These oaths articulate the ethical responsibilities and societal role inherent in the practice of veterinary medicine.

### 3.2 Quality

The International Organization for Standardization standard ISO 9000:2015 defines “quality” as the degree to which a set of inherent characteristics of an object fulfills specified requirements (29). Within this framework, an “object” may also be conceptualized as a service. This definition implies the existence of two interrelated variables: first, the inherent characteristics of the object or service itself; and second, the requirements of the recipient. Both variables are dynamic and may vary across time and place.

The World Organisation for Animal Health, in its Terrestrial Animal Health Code, stipulates that national Veterinary Services—defined as the collective of governmental and non-governmental actors responsible for implementing animal health and welfare measures—should adhere to a set of interdependent principles to ensure the quality of their activities (30). These principles include professional judgement, independence, objectivity, impartiality, integrity, transparency, a scientific basis, and intersectoral collaboration.

Furthermore, with regard to personnel, the Code specifies that individuals should possess the requisite qualifications, expertise, and experience to ensure competence in making sound professional judgements. It also emphasizes the necessity of safeguarding personnel from undue pressures that could compromise their professional judgement or decision-making processes.

If these WOAH-defined requirements for Veterinary Services and their personnel are interpreted as inherent characteristics of veterinarians that are pertinent to the quality of their professional performance, these characteristics may be analytically categorized into three domains: (i) veterinary scientific and technical competencies, (ii) moral and ethical points, and (iii) behavioral competencies.

#### 3.2.1 Veterinary scientific and technical aspects

The first category of requirements pertains to the scientific foundations of veterinary medicine, as well as to the essential knowledge, competencies, and expertise expected of the veterinarian. This encompasses adherence to current scientific evidence and the prevailing standards of technical practice. The basis for these competencies is established during undergraduate veterinary education. Following graduation, veterinarians are expected to sustain and further advance their knowledge and skills through ongoing professional development and lifelong learning.

#### 3.2.2 Moral aspects

Veterinary medicine inevitably involves ethical dilemmas, as the interests of animals, owners, and society often conflict rather than align. Veterinarians must be able to recognize and evaluate the values at stake, and balance them in a professional, fair, and well-justified manner. It is essential that their judgement remains independent and free from external pressures that could compromise their decisions. Throughout their careers, veterinarians should stay open to new insights and evolving societal expectations. Engaging in discussions with colleagues and stakeholders can support sound ethical decision-making.

#### 3.2.3 Behavioral aspects

The third group of characteristics concerns behavioral aspects, including honesty, integrity, reliability, courtesy, accuracy, punctuality, and availability. It is not sufficient to merely know what veterinary actions should be taken; these actions must also be carried out carefully and effectively in practice.

Both the second (moral) and third (behavioral) groups relate to elements commonly addressed in professional codes of conduct or deontological codes. Typically developed by the profession itself, these codes establish standards for ethical behavior and professional practice. They aim to uphold core principles such as professional independence, confidentiality, honesty, integrity, and dignity (31).

In several countries, the commitment to these quality aspects is formally expressed through an oath taken by veterinary graduates. For example, the oath used in the Netherlands states: “I swear/promise that I will practice veterinary medicine to the best of my ability, serving animals, my fellow human beings, and society […] I prioritize the interests of the animal and take into account the views of the person responsible for the animal, while respecting the intrinsic value of the animal […] I will handle my veterinary knowledge with integrity and act accordingly […]” (32).

## 4 Quality assurance in veterinary medicine

### 4.1 Preparing to start: pre-graduate education and developing starting competences

The foundation of high-quality veterinary services is established during pre-graduate training, where candidates acquire the knowledge and skills necessary to achieve the so-called “day-one” or starting competences required of the Omni-competent veterinarian. This concept refers to a veterinary graduate who is adequately prepared to enter professional practice and meets the requirements for licensure across all fields of veterinary medicine (33). However, in light of the continuous expansion of veterinary science and technological innovation, the feasibility of maintaining such Omni-competence is increasingly being questioned (34).

The European Union has played a key role in promoting and maintaining high standards of veterinary education across its Member States. The creation of the European Economic Community (EEC) in 1957 (Treaty of Rome) marked an important step toward a common market based on the free movement of goods, persons, services, and capital (35). Within this framework, efforts were made to support the mobility of veterinarians and the provision of veterinary services. These were formalized through two Council Directives (78/1026/EEC and 78/1027/EEC) and one Council Decision (78/1028/EEC), which ensured mutual recognition of veterinary diplomas and coordinated minimum training requirements (36–38). The Decision also established the Advisory Committee on Veterinary Training (ACVT) to help maintain high educational standards.

The ACVT concluded that a consistent standard could best be achieved through a permanent, Europe-wide evaluation system for veterinary training institutions. Responsibility for this peer-review system was given to the European Association of Establishments for Veterinary Education (EAEVE). In 2000, governance was transferred to a Joint Education Committee of EAEVE and the Federation of Veterinarians of Europe (FVE) under the Thessaloniki Agreement (39).

This led to the European System of Evaluation of Veterinary Training (ESEVT), which plays a central role in quality assurance in veterinary education. The system is unique in veterinary education and includes 69 EU institutions, 63 of which have received positive evaluations (40). It has also expanded beyond the EU and is accredited by the European Association for Quality Assurance in Higher Education (79).

Although originally designed to support the internal market, especially professional mobility, the legislation, and ESEVT have significantly improved veterinary education standards across Europe, particularly by strengthening less developed institutions.

### 4.2 Maintaining quality throughout the veterinary career: regulation of the profession

Mastery of the required entry-level competencies is a fundamental condition for admission to the veterinary profession; however, it does not, in itself, guarantee the sustained maintenance of professional competence throughout an individual's career. Advances in veterinary science occur rapidly, rendering knowledge and practices that are deemed adequate at one point potentially insufficient in the future. In this context, Continuous Professional Development (CPD) is indispensable.

Furthermore, the mere possession of appropriate competencies does not ensure their effective application, nor does it guarantee that veterinarians consistently deliver services of the requisite quality. To safeguard public trust in the profession, many countries have therefore established regulatory frameworks governing veterinary practice. Such regulation typically entails that access to the practice of veterinary medicine is contingent upon the fulfillment of specific conditions. Authorization or licensure is granted only to those who meet these requirements and may be revoked in cases of non-compliance.

The regulation of the veterinary profession is generally build on a range of instruments, including:

- the establishment of standards and procedures for initial licensure;
- the development of requirements and procedures for re-licensure, including both the quality and quantity of CPD activities;
- the accreditation of qualifications awarded by institutions providing veterinary education and training at both undergraduate and postgraduate levels;
- the maintenance of publicly accessible registers of licensed practitioners;
- the adoption of codes of deontology and professional conduct guidelines;
- the investigation of alleged breaches of professional standards and, where appropriate, the imposition of disciplinary measures, including the withdrawal of licensure.

Individually, these instruments have limited impact; however, when implemented collectively, they constitute a coherent and effective system for ensuring and enforcing quality in veterinary medicine. For optimal functioning, these instruments should be unified under a single authority with governance capable of overseeing the system and managing their interrelationships.

### 4.3 Implementation by the Ministry or, after delegation, by the Veterinary Statutory Body

In general, the regulation of the veterinary profession is initiated by a competent public authority, most commonly the Ministry of Public Health or the Ministry of Agriculture. The implementation of such regulation is undertaken either directly by the authority itself or delegated to a professional body, such as an Order, Council, or Chamber, collectively referred to as a Veterinary Statutory Body (VSB). In systems of self-regulation, the state grants statutory authority to the professional body through legislation.

Among the 37 countries represented within the Federation of Veterinarians of Europe (FVE), including all European Union Member States, 22 have established a VSB and 15 have not (see Table 2) (41, 42). Within the European Union specifically, 16 out of 27 Member States have instituted a VSB, whereas 11 have not. In certain countries—such as Germany, Poland, Portugal, Romania, Spain, and Italy—where VSBs operate at a regional level, multiple bodies exist within a single national jurisdiction.

**Table 2:** List of Veterinary Statutory Bodies in European countries (EU-countries and non-EU countries) represented in the Federation of Veterinarians of Europe.

EU-country	Veterinary statutory body
Austria	Österreichische Tierärztekammer
Belgium	Orde der Dierenartsen/Ordre des Vétérinaires
Bulgaria	No Veterinary Statutory Body
Croatia	Hrvatska Veterinarska Komora
Cyprus	No Veterinary Statutory Body
Czechia	Komora veterinárních lékaru Ceské republiky
Denmark	No Veterinary Statutory Body
Estonia	No Veterinary Statutory Body
Finland	No Veterinary Statutory Body
France	Ordre des Vétérinaires
Germany	BundesTierärztekammer/ Arbeitsgemeinschaft der Deutschen Tierärztekammern
Greece	No Veterinary Statutory Body
Hungary	Magyar Allatorvoski Kamara
Ireland	Veterinary Council of Ireland
Italy	Federazione Nazionale Ordini Veterinari Italiani
Latvia	No Veterinary Statutory Body
Lithuania	No Veterinary Statutory Body
Luxembourg	Collège Vétérinaire du Grand-Duché de Luxembourg
Malta	No Veterinary Statutory Body
Netherlands	No Veterinary Statutory Body
Poland	Krajowa Izba Lekarsko-Weterynaryjna
Portugal	Ordem dos Médicos Veterinarios
Romania	The Romanian College of Veterinary Surgeons
Slovak Republic	Komora veterinárnych lekárov Slovenskej republiky
Slovenia	Veterinarska zbornica Slovenije
Spain	Consejo General de Colegios Oficiales de la Profesión Veterinaria de España
Sweden	No Veterinary Statutory Body
Non-EU country	Veterinary statutory body
Albania	Albanian Chamber of Veterinary Surgeons
Armenia	No Veterinary Statutory Body
Bosnia-Herzegovina	Veterinary Chamber of the Republic of Srpska
Iceland	No Veterinary Statutory Body
Montenegro	Veterinary Chamber of Montenegro
North Macedonia	Veterinary Chamber of the Republic of North Macedonia
Norway	No Veterinary Statutory Body
Serbia	Veterinarske komore Srbije
Switzerland	No Veterinary Statutory Body
United Kingdom	Royal College of Veterinary Surgeons

In Estonia, an organization known as the Estonian Veterinary Chamber was established in 2024. Despite its designation as a “chamber,” which typically implies the status of a VSB, the organization exhibits characteristics more consistent with those of a professional association rather than a statutory regulatory body.

The World Organisation for Animal Health (WOAH) places considerable emphasis on the role and functioning of Veterinary Statutory Bodies (VSBs) in regulating the veterinary profession across its member countries. This is reflected in key instruments such as the *Terrestrial Animal Health Code* and the Performance of Veterinary Services (PVS) Pathway, which is used to evaluate national veterinary services (43).

Although VSBs broadly share common objectives, their modes of operation vary significantly (44). In this respect, they resemble other professional organizations which, particularly in the United Kingdom context, often function as composite entities combining elements of regulatory authorities, learned societies, and professional associations (45).

In 2016, the Federation of Veterinarians of Europe introduced a VSB self-assessment scheme, which generated 19 responses (46). The assessment covered a range of topics, including legislative frameworks, licensing and registration procedures, minimum standards for education, governance structures, and funding mechanisms. The results revealed substantial variation among countries. For instance, only a limited number of countries—such as Croatia, Hungary, Ireland, the Netherlands, and the United Kingdom—extend registration requirements beyond veterinarians to include veterinary nurses and technicians. While registers are publicly accessible in most countries, this is not universally the case.

Furthermore, although nearly all participating countries have established a Veterinary Code of Conduct, adherence to such codes is not always mandatory. Their practical impact varies considerably, ranging from situations where the profession is largely unaware of the code to contexts in which its principles are enforced. Similar divergences are evident in other areas examined by the self-assessment, including involvement in pre-graduate education, requirements for continuing professional development, and the scope of authority exercised by VSBs.

A defining characteristic of VSBs is the requirement for mandatory membership. However, there are notable differences in the categories of veterinary professionals subject to regulation. While veterinarians engaged in clinical practice are consistently regulated across all VSBs, the inclusion of other categories varies between jurisdictions. For example, in Belgium, veterinarians employed as permanent government officials are exempt from mandatory registration when performing veterinary acts in their official capacity (47). By contrast, in Netherlands, a country without a VSB, veterinarians employed as permanent officials—such as those working for the national food safety authority—remain subject to the jurisdiction of the Veterinary Disciplinary Board (48).

## 5 Changing world and potential impact on the quality of veterinary medicine.

For many years, Veterinary Statutory Bodies (VSBs) have exercised considerable autonomy in regulating the veterinary profession in accordance with established norms and practices. However, the context in which veterinary medicine is practiced has undergone significant transformation, with changes occurring at an increasingly rapid pace. Importantly, many of these developments are interrelated and have the potential to substantially affect the quality and integrity of veterinary practice.

One of the most notable developments is the expansion of free-market principles, including liberalization and deregulation, particularly within the European Union, where there is a strong emphasis on competition and market efficiency. Parallel to this, structural changes have emerged in the ownership, liability, financing, and organization of veterinary practices. Notably, there has been a rise in corporate veterinary groups and chain practices owned by external investors. These entities often operate with different financial priorities and shorter investment horizons compared to traditional veterinary practitioners. As a result, increasing market pressures and deregulation may challenge the position of veterinarians as independent and impartial professionals.

Another significant shift concerns evolving societal expectations regarding the role of veterinarians, particularly in relation to the human–animal relationship. Perceptions of animal welfare have changed markedly over time. Whereas, animal welfare was once primarily associated with physical health and productivity, contemporary perspectives place greater emphasis on the promotion of positive welfare states, beyond merely the avoidance of suffering. This shift is strongly reflected in the standards and guidance of the World Organisation for Animal Health (49). Although legal responsibility for animal welfare rests with the owner, veterinarians are increasingly expected to provide well-founded, ethically informed advice.

In addition, the profession is being shaped by a range of technological and demographic developments. These include the increasing use of automation, the emergence of artificial intelligence, and the expansion of telemedicine, all of which are transforming modes of service delivery. At the same time, notable changes in the demographic composition of the veterinary workforce are influencing professional dynamics and expectations.

Collectively, these developments underscore the need for VSBs to continuously adapt their regulatory approaches in order to ensure the continued quality, independence, and societal relevance of veterinary practice.

### 5.1 Free market and deregulation

First and foremost, significant emphasis has been placed on the proper functioning of the market. As noted, the European Economic Community (EEC) aimed to establish a common market based on the free movement of goods, persons, services, and capital—an objective reaffirmed in 1992 with the Treaty on European Union (50). This led to the EU's competition law framework, designed to prevent distortions such as cartels, abuses of dominant positions, monopolistic mergers, and state aid.

Within this context, existing regulatory practices in veterinary medicine—such as those concerning fee structures and the geographical establishment of new practices—were reassessed. Where such practices were deemed to constitute unjustified restrictions on market freedom, they were abolished. Consequently, in most EU Member States, rules and guidelines establishing (minimum) fees were discontinued.

An exception persists in Germany in the form of the Veterinary Fees Regulation (Tierärztegebührenordnung, GOT) (51). At first glance, this regulation appears to conflict with European competition law. However, the Court of Justice of the European Union has clarified (e.g., Case C-35/99, Arduino) that competition law applies to agreements between undertakings, and not to regulatory measures imposed by the state (52). In Germany, the GOT is established by the Federal Ministry of Food and Agriculture, and therefore does not fall within the scope of prohibited agreements.

From the late 20th century into the early 21st century, the European Union devoted increasing attention to the regulation of the liberal professions. Established customs and regulatory frameworks governing veterinary medicine were subjected to critical scrutiny, particularly with regard to their compatibility with the principles of market efficiency and competition (53).

Directive 2006/123/EC on services in the internal market further defined the conditions under which authorization schemes may be maintained, requiring justification by overriding public interest and proportionality (54). In veterinary medicine, such conditions are generally met due to the importance of safeguarding animal and public health, where ex post controls are often insufficient.

The Directive also establishes criteria for granting individual authorizations, which must be non-discriminatory, objective, transparent, and proportionate. More recently, with the rise of corporate veterinary groups, competition authorities have shifted focus toward the availability and accessibility of services.

Finally, closely linked to this growing market orientation are international trade agreements and their implications, particularly with regard to animal health standards, including (non-) vaccination policies. These developments have, in some cases, led to practices such as the long-distance transport of live animals and the large-scale culling of healthy livestock during disease outbreaks such as Foot and Mouth Disease which may conflict with veterinary ethical standards.

### 5.2 Changing expectations toward veterinarians

Quality may be conceptualized as comprising two interrelated dimensions: the inherent characteristics of the service provided and the expectations of its users. Within veterinary medicine, these expectations have undergone significant transformation, largely driven by evolving societal values and shifts in the human–animal relationship, thereby elevating the standards to which veterinarians are held.

In recent decades, the intensification of animal husbandry, coupled with advances in the study of animal behavior, has contributed to increased societal concern for animal welfare and stimulated critical reassessment of traditional practices (55). This evolution is reflected in the progressive institutionalization of animal welfare at multiple governance levels. At the international level, the World Organisation for Animal Health (WOAH) identified animal welfare as a strategic priority in 2001 and has since developed a comprehensive body of standards, subsequently incorporated into its Terrestrial Animal Health Code (56, 57).

At the European Union level, animal welfare has been increasingly embedded within the legal framework, culminating in the explicit recognition of animals as sentient beings under the Treaty of Lisbon and the corresponding obligation to integrate animal welfare considerations into policymaking processes. Nationally, both the Netherlands and Belgium have strengthened their legal approaches. Whereas, the Netherlands situates the recognition of animals as sentient beings within specific legislative instruments, Belgium has adopted a more expansive approach, including the classification of animals as “quasi-goods” and the constitutional entrenchment of animal protection in 2024 (58).

These developments have significant implications for veterinary practice. Animal welfare has taken on a more prominent role in veterinary education, and veterinarians are increasingly required to navigate between their professional responsibilities and the ultimate decision-making authority of animal owners (59).

At the same time, the profession has shifted—particularly in large animal practice—from a curative to a preventive model, focusing on biosecurity, nutrition, housing, and vaccination. In line with this paradigm shift, the prophylactic use of antimicrobials is, with very limited exceptions, prohibited under Regulation (EU) 2019/6 on veterinary medicinal products (60). However, preventive measures often lack immediate visibility and depend on owner compliance, which can limit their perceived value despite long-term cost-effectiveness (61–63).

In parallel, veterinarians are increasingly expected to act as “gatekeepers” in the implementation of animal health policies, particularly regarding responsible use of veterinary medicines (64, 65). This role introduces additional complexities, including potential tensions between advisory, supervisory, and reporting responsibilities. In such cases, professional obligations—particularly with respect to animal welfare—may take precedence over client loyalty (66).

### 5.3 Corporates

Traditionally, veterinary practices have been owned and managed by individual practitioners or small partnerships, who assume direct responsibility for the financial performance, liabilities, and overall management of the practice (67). In recent years, however, many countries have witnessed a marked increase in corporate ownership within the veterinary sector. Such corporate structures typically consist of legal entities that operate multiple practices, employ veterinarians, and are often managed by non-veterinary professionals. Within this model, shareholders are generally not personally liable for corporate debts and derive financial returns through dividends and capital appreciation (19).

Empirical data illustrate the extent of this transition. A recent survey covering 37 European countries indicates that 16% of veterinarians are employed in corporate practices, compared to 51% in independent practices and 33% in alternative ownership structures (68). The prevalence of corporate employment is particularly pronounced in countries such as the United Kingdom, Sweden, and Norway. In the United Kingdom, for instance, the proportion of veterinarians working within corporate practices has increased substantially, with corporate ownership in companion animal practice rising from 10% in 2013 to approximately 60% in 2024 (69).

This process of corporatization and consolidation has prompted considerable debate regarding its implications for veterinary practice. Key concerns relate to the potential impact on professional independence, the emergence of conflicts of interest, and the accessibility of veterinary services. From the perspective of service quality, corporatization presents a complex interplay of advantages and disadvantages. On the one hand, corporate governance structures, standardized protocols, and internal performance targets may constrain veterinarians' professional autonomy and influence clinical decision-making.

On the other hand, corporatization can generate significant efficiencies through economies of scale. It may facilitate investment in advanced infrastructure, diagnostic technologies, and innovation, while also expanding opportunities for professional development and continuing education within an international context. Moreover, such structures can promote differentiation and specialization, potentially enhancing the scientific and technical quality of veterinary care (see Table 3).

**Table 3:** Opportunities and challenges associated with corporatization in veterinary medicine (19).

Opportunities	Challenges
Economics of scale: cost of efficiency and resource sharing, tax optimization, and bulk purchase discounts.	Volatility of economic business decisions: neglect less profitable areas of veterinary medicine, e.g., rural and remote areas, and less profitable species groups, such as livestock.
Central support for administrative and human resources assistance.	Monopoly-risks: reduced competition in certain regions or local markets.
Advanced veterinary treatments: more specialists, specialized equipment, and data collection.	Increased prices: costs rising faster than inflation also due to (the advertisement of) more advanced treatments and intervention protocols.
More opportunities for career development, training, mentoring, professional growth.	Decision-making constraints: risk of facing limitations in clinical and operational decision-making processes due to the in-house financial requirements/policies of the corporate.
	Profit focus: potential overemphasis on profitability and competitive practices contrary to public policy and/or the veterinary code of conduct.

Nevertheless, questions remain as to whether the increased availability of diagnostic and therapeutic interventions always serves the best interests of animals and their owners. The availability of such services may itself stimulate demand, potentially leading to the provision of care that exceeds what is strictly necessary. In addition, these interventions are often associated with substantial costs, which may limit accessibility for certain client groups.

These issues have attracted growing attention from policymakers and regulatory bodies. For example, studies commissioned by the Dutch government have examined pricing developments in companion animal practice. Additional studies made comparative analyses of regulatory influences on tariffs across European Union Member States (70, 71). Comparable investigations have been undertaken by the Belgian and French competition authorities, reflecting broader concerns regarding price dynamics within the sector.

Finally, corporatization may also have implications for the geographical distribution of veterinary services. Investment strategies that prioritize profitability can lead to a concentration of practices in urban or economically attractive regions, potentially resulting in reduced service provision in rural or less densely populated areas (72).

### 5.4 Artificial intelligence

A significant technological development increasingly shaping veterinary medicine is the emergence and integration of artificial intelligence (AI) and its diverse applications. AI offers substantial potential advantages, particularly in its capacity to process and analyze large volumes of data with exceptional speed and efficiency. In clinical settings, AI can be applied to tasks such as the interpretation of radiographic images, the assessment of gait patterns, and the evaluation of pain-related facial expressions, among others. These applications may support clinical decision-making, particularly in cases that fall within well-defined parameters. In addition, AI models can be trained to classify disease stages and generate prognostic assessments. In certain contexts, such systems may surpass human practitioners in terms of speed, accuracy, and cost-effectiveness. Beyond clinical practice, AI may also enhance administrative efficiency by facilitating appointment scheduling, client communication, and workflow optimization (73).

Notwithstanding these advantages, the integration of AI into veterinary medicine necessitates a cautious and critical approach. AI is limited to narrow, task-specific intelligence, which means it can perform only a certain set of actions based on its programming (74). The reliability of AI outputs is highly dependent on system design, including the quality, scope, and representativeness of the underlying data. It is therefore imperative that veterinarians possess a sufficient level of understanding of these technologies and retain ultimate responsibility for clinical decision-making. Continuous evaluation of the relevance, performance, and limitations of AI applications is essential to ensure that their deployment results in meaningful improvements in patient care (75, 76).

The adoption of AI further raises a range of ethical considerations. Core principles of medical ethics—non-maleficence, beneficence, respect for autonomy, and justice (77)—remain directly applicable in this context. Concerns regarding accuracy and reliability are particularly salient: while well-designed systems may enhance patient outcomes, flawed or insufficiently validated tools carry the risk of causing harm. Importantly, performance under controlled testing conditions does not necessarily translate into equivalent reliability in real-world clinical environments, highlighting the need for representative training and validation datasets.

Additional ethical challenges include the risk of overdiagnosis, for example through the indiscriminate application of AI tools to healthy populations, as well as issues relating to transparency, data security, and accountability (78).

Furthermore, the increasing accessibility of veterinary information via digital platforms is reshaping the relationship between veterinarians and animal owners. Practitioners are increasingly required to engage with well-informed clients and to address more complex and detailed inquiries regarding diagnosis and treatment options. In some instances, animal owners may rely on AI-based tools to conduct preliminary assessments or initiate interventions prior to seeking professional veterinary advice.

Given the rapid pace of technological advancement, AI has the potential to exert a significant influence on the quality of veterinary services, both positively and negatively. This underscores the necessity for regulatory frameworks to evolve in parallel with technological developments.

The issues discussed above represent only a subset of the broader transformations affecting veterinary practice. Although a comprehensive analysis falls beyond the scope of this discussion, the central question remains the extent to which such developments impact the quality of veterinary services and whether existing regulatory systems are adequately equipped to respond to these challenges.

## 7 Preliminary conclusions and further questions

Over time, veterinary medicine has developed into a science- and evidence-based profession whose impact extends well beyond individual animals and their owners to encompass broader societal interests. The quality of veterinary services must therefore be regarded as a matter of public concern. Regulatory frameworks are not merely desirable but essential to safeguard both animal and public health. They have been established to protect individuals who may lack the expertise to adequately assess the quality of veterinary care.

However, a first inspection of regulatory approaches across European countries reveals a fragmented and inconsistent landscape. There is no uniform understanding of what constitutes veterinary practice. Jurisdictions diverge significantly in defining the scope of reserved acts and the roles of veterinary paraprofessionals. Similarly, the categories of professionals subject to regulation vary considerably. The implementation of regulatory frameworks is equally inconsistent: while some countries rely on direct governmental oversight, others delegate substantial authority—within statutory limits—to Veterinary Statutory Bodies (VSBs) established by professional associations. Requirements for re-licensing and continuing professional development differ markedly, as do codes of conduct and deontological standards. This heterogeneity raises concerns regarding the coherence and effectiveness of veterinary regulation across Europe.

Further inconsistencies arise in the mandates and capacities of VSBs. In some jurisdictions, these bodies are confined to regulatory oversight, whereas in others they combine regulatory functions with member services and the representation of professional interests, potentially giving rise to conflicts of role and purpose. Disparities in human resources and legal expertise further deepen differences in regulatory performance, calling into question the overall robustness of existing systems.

At the same time, veterinary medicine is undergoing profound and rapid transformation. Processes of deregulation and market liberalization, shifting societal expectations—particularly in relation to the human–animal relationship—and the accelerated development of artificial intelligence are fundamentally reshaping the profession. These changes are not peripheral; they directly affect both the position of the veterinarian and the quality of veterinary services.

Against this backdrop, it is no longer sufficient to assume that existing regulatory systems remain fit for purpose. On the contrary, there is a need to critically assess whether they continue to fulfill their intended functions in an effective and efficient manner. In particular, questions must be raised as to whether regulators are sufficiently proactive in adapting to evolving circumstances, whether they employ rigorous quality assurance mechanisms to monitor and improve their performance, and whether current practices reflect identifiable best standards. The absence of clear and consistent answers to these questions underscores the urgent need for systematic comparative research and for a thorough evaluation of the impact of regulatory frameworks on both service quality and the evolving role of the veterinary profession.

## References

[1] Jones S Koolmees P. Animal healing in sacred societies, 1500–1700. In: A Concise History of Veterinary Medicine. Cambridge: Cambridge University Press (2022). p. 13–38.

[2] Steele J. Veterinary public health: past success, new opportunities. Prev Vet Med. (2008) 86:224–43. doi: 10.1016/j.prevetmed.2008.02.014 18417229 PMC7132490

[3] McBride E Baugh S. Animal welfare in context: historical, scientific, ethical, moral and one welfare perspectives. In: Human/Animal Relationships in Transformation: Scientific, Moral and Legal Perspectives. Cham, Switzerland: Palgrave Macmillan (2022). p. 119–47.

[4] Berckmans D. Precision livestock farming technologies for welfare management in intensive livestock systems. Rev Sci Tech. (2014) 33:189–96. doi: 10.20506/rst.33.1.2273 25000791

[5] Upinder K Malacco V Bai H Price T Datta A Xin L . Invited review: integration of technologies and systems for precision animal agriculture—a case study on precision dairy farming. J Anim Sci. (2023) 101:skad206. doi: 10.1093/jas/skad206 37335911 PMC10370899

[6] Bao J Xie Q. Artificial intelligence in animal farming: a systematic literature review. J Clean Prod. (2022) 331:129956. doi: 10.1016/j.jclepro.2021.129956

[7] Sundberg A. Floods, Worms, and Cattle Plague: Nature-Induced Disaster at the Closing of the Dutch Golden Age, 1672–1764. [Dissertation] (2015). University of Kansas, Lawrence, KS, United States.

[8] Smith DF. Lessons of history in veterinary medicine. J Vet Med Educ. (2013) 40:2–11. doi: 10.3138/jvme.1112.04 23470241

[9] King LJ Ferguson J Galligan D Salman M Osburn BI. One Health, food security, and veterinary medicine. J Am Vet Med Assoc. (2013) 242:739–43. doi: 10.2460/javma.242.6.739 23445280

[10] Robin D. Bourgelat et les écoles vétérinaires. Bull Soc Fr Hist Méd Sci Vét. (2002) 1:34. doi: 10.3406/bhsv.2002.1204

[11] Jones S Koolmees PA. Spread of veterinary education institutions around the globe (List of the first veterinary school established in selected nations, 1762–1960s). In: *A Concise History of Veterinary Medicine*. New Approaches to the History of Science and Medicine. Cambridge: Cambridge University Press (2022). p. 375–7.

[12] Saunders LZ. Virchow's contributions to veterinary medicine: celebrated then, forgotten now. Vet Pathol. (2000) 37:199–207. doi: 10.1354/vp.37-3-199 10810984

[13] Koolmees P. Belgische studenten van 's Rijks Veeartsenijschool in Utrecht, 1821–1830. Argos. (2018) 58:285–93. Available online at: https://veterinaryhistory.nl/wp-content/uploads/2021/01/452573_argos_58.pdf (Accessed May 26, 2026).

[14] United Kingdom. Veterinary Surgeons Act 1881. (1881). Available online at: https://vlex.co.uk/vid/veterinary-surgeons-act-1881-808050365 (Accessed December 9, 2025).

[15] United Kingdom. Veterinary Surgeons Act 1948. (1948). Available at: https://www.legislation.gov.uk/ukpga/1948/52/pdfs/ukpga_19480052_en.pdf (Accessed December 9, 2025).

[16] Netherlands. Ontwerp van wet tot regeling van de uitoefening der veeartsenijkunst (Law of 8 July 1874 regulating the practice of veterinary medicine). (1874). Available online at: https://repository.overheid.nl/frbr/sgd/18731874/0000414735/1/pdf/SGD_18731874_0000717.pdf (Accessed May 26, 2026).

[17] Belgium. Law of 28 August 1991 on the practice of veterinary medicine. Belgisch Staatsblad, 22981. (1991). Available online at: https://www.ejustice.just.fgov.be/mopdf/1991/10/15_1.pdf (Accessed December 9, 2025).

[18] European Union. Regulation (EU) 2016/429 of the European Parliament and of the Council of 9 March 2016 on transmissible animal diseases and amending and repealing certain acts in the area of animal health (“Animal Health Law”). Official Journal of the European Union. (2016). p. 59. Available online at: https://eur-lex.europa.eu/legal-content/EN/TXT/?uri=OJ:L:2016:084:TOC (Accessed May 26, 2026).

[19] Diana A Mickov D van Dobbenburgh R Jansen W Sternberg-Lewerin S Neumann S . Quo vadis: is corporatisation reshaping companion animal veterinary care in Europe? Pets. (2025) 2:15. doi: 10.3390/pets2010015

[20] Federation of Veterinarians of Europe. Code of Conduct and Definition of Veterinary Act. (2019). Available online at: https://fve.org/cms/wp-content/uploads/FVE_Code_of_Conduct_2019_R1_WEB.pdf (Accessed May 26, 2026).

[21] Federation of Veterinarians of Europe. Definition of the Veterinarian. (2012). FVE archives document FVE/12/docs/008a_rev10.

[22] Netherlands. Wet van 19 mei 2011, houdende een integraal kader voor regels over gehouden dieren en daaraan gerelateerde onderwerpen (Wet dieren). Staatsblad van het Koninkrijk der Nederlanden. (2011) 345:1–63. Available online at: https://zoek.officielebekendmakingen.nl/stb-2011-345.pdf (Accessed December 8, 2025).

[23] Arendt H. The Human Condition, 2nd Edn. Chicago: The University of Chicago Press (1958). p. 91.

[24] Lamon H. Hoe vrij is het vrij beroep nog? Beschouwingen door de bril van een advocaat. Heverlee: LeA Uitgevers (2022).

[25] Court of Justice of the European Union. Urbing-Adam, 11 October 2001, Case C-267/99, ECR 2001, I-7467. (2001). Available online at: https://eur-lex.europa.eu/legal-content/EN/TXT/HTML/?uri=CELEX:61999CJ0267 (Accessed May 26, 2026).

[26] European Union. Directive 2005/36/EC of the European Parliament and of the Council on the recognition of professional qualifications. Official Journal of the European Union, L 255, 30 September (2005). p. 22. Available online at: https://eur-lex.europa.eu/legal-content/EN/TXT/PDF/?uri=CELEX:32005L0036 (Accessed May 26, 2026).

[27] Ordre National des Vétérinaires. Serment de Bourgelat. (n.d.). Available online at: https://www.veterinaire.fr/system/files/files/2021-12/293_o-pre-bourgelat_p1.pdf (Accessed May 26, 2026).

[28] Royal College of Veterinary Surgeons. Declaration on admission to the profession. (n.d.). Available online at: https://www.rcvs.org.uk/veterinary-professionals/conduct-and-guidance/code-of-professional-conduct-for-veterinary-surgeons (Accessed May 26, 2026).

[29] International Organization for Standardization. ISO 9000: Quality management systems — Fundamentals and vocabulary. (2015). Available online at: https://www.iso.org/obp/ui/en/#iso:std:iso:9000:ed-4:v1:en (Accessed December 8, 2025).

[30] World Organisation for Animal Health (WOAH). Terrestrial Animal Health Code. (2019). Available online at: https://rr-europe.woah.org/app/uploads/2020/08/oie-terrestrial-code-1_2019_en.pdf (Accessed July 29, 2025).

[31] European Commission. Directorate-General for Internal Market, Industry, Entrepreneurship and SMEs. In: Handbook on the Implementation of the Services Directive. Luxembourg: Publications Office of the European Union (2022). Available online at: https://op.europa.eu/en/publication-detail/-/publication/60e2d020-6c6f-11ed-9887-01aa75ed71a1 (Accessed May 26, 2026).

[32] Netherlands. Oath for newly graduated veterinarians in the Netherlands. (n.d.). Available online at: https://students.uu.nl/sites/default/files/dgk_eed_-_belofte_voor_de_dierenarts_mrt_2019.pdf (Accessed August 4, 2025).

[33] European Coordinating Committee on Veterinary Training. Recommendations to manage expectations around the value of Day One Competences. (2015). Available online at: https://eccvt.fve.org/cms/wp-content/uploads/ECCVT-position-on-D1C_adopted_April2025.pdf (Accessed May 26, 2026).

[34] Bok H. Samen leren opleiden. Tijdschr Diergeneeskd. (2025) 150:15–7.

[35] European Economic Community. Traité instituant la Communauté Économique Européenne. (1957). Available online at: https://eur-lex.europa.eu/legal-content/FR/TXT/PDF/?uri=CELEX:11957E/TXT (Accessed May 26, 2026).

[36] European Economic Community. Council Directive 78/1026/EEC concerning the mutual recognition of diplomas, certificates and other evidence of formal qualifications in veterinary medicine, including measures to facilitate the effective exercise of the right of establishment and freedom to provide services, OJ L 362, 23 December (1978). p. 1–6. Available online at: https://eur-lex.europa.eu/legal-content/FR/TXT/HTML/?uri=CELEX:31978L1026 (Accessed May 26, 2026).

[37] European Economic Community. Council Directive 78/1027/EEC on the coordination of laws, regulations and administrative provisions relating to the activities of veterinary surgeons, OJ L 362, 23 December. (1978). p. 7–9. Available online at: https://eur-lex.europa.eu/legal-content/EN/TXT/PDF/?uri=OJ:L:1978:362:FULL (Accessed May 26, 2026).

[38] European Economic Community. Council Decision 78/1028/EEC of 18 December 1978 setting up an Advisory Committee on Veterinary Training, OJ L 362. (1978). p. 10–11. Available online at: https://eur-lex.europa.eu/legal-content/EN/TXT/PDF/?uri=OJ:L:1978:362:FULL (Accessed May 26, 2026).

[39] Federation of Veterinarians of Europe and European Association of Establishments for Veterinary Education. (2000). Thessaloniki Agreement. Brussels: FVE Archive.

[40] European Association of Establishments for Veterinary Education. European System of Evaluation of Veterinary Training: Status of EAEVE Veterinary Education Establishments. (2025). Available online at: https://www.eaeve.org/fileadmin/downloads/establishments_status/ESEVT_Status_of_EAEVE_Establishments_Sept2025.pdf (Accessed May 26, 2026).

[41] Federation of Veterinarians of Europe. List of Regulators and Professional Associations. (2022). Available online at: https://fve.org/cms/wp-content/uploads/List-of-regulators_CAs_2022-1.pdf (Accessed August 7, 2025).

[42] Federation of Veterinarians of Europe. Veterinary Regulators in Europe: update and recommendations. Brussels: FVE Archives (2023).

[43] World Organisation for Animal Health (WOAH). Tool for the Evaluation of Performance of Veterinary Services (PVS Tool). (2019). Available at: https://www.woah.org/fileadmin/Home/eng/Support_to_OIE_Members/docs/pdf/2019_PVS_Tool_FINAL.pdf (Accessed May 26, 2026).

[44] Vaarten J. Veterinary statutory bodies in Europe. In: Proceedings of the 3rd OIE Global Conference on Veterinary Education and the Role of Veterinary Statutory Bodies, Foz do Iguaçu, Brazil. (2014).

[45] Friedman A Afitska N. Professional bodies. J Prof Organ. (2023) 10:1–15. doi: 10.1093/jpo/joad001

[46] Federation of Veterinarians of Europe. FVE Self-assessment Scheme for Veterinary Statutory Bodies. Brussels: FVE Archives (Ref: FVE/2016/doc/021). (2016).

[47] Belgium. Wet op de uitoefening van de diergeneeskunde, 28 August 1991, Art. 4. (1991). Available online at: https://www.ejustice.just.fgov.be/eli/wet/1991/08/28/1991016144/justel (Accessed May 26, 2026).

[48] Veterinair Beroepscollege. Case VB 2020/09, ECLI:NL:TDIVBC:2020. Brussels (2020). p. 9.

[49] World Organisation for Animal Health (WOAH). Animal welfare: a vital asset for a more sustainable world. Paris: WOAH (2024).

[50] European Union. Treaty of Maastricht on European Union. (1992). Available online at: https://eur-lex.europa.eu/EN/legal-content/summary/treaty-of-maastricht-on-european-union.html (Accessed May 26, 2026).

[51] Germany. Tierärztegebührenordnung (GOT) (2022). Available online at: https://www.gesetze-im-internet.de/got_2022/BJNR140100022.html (Accessed May 26, 2026).

[52] Court of Justice of the European Union. Case C-35/99. Arduino (2000). Available online at: https://curia.europa.eu/juris/liste.jsf?num=C-35/99 (Accessed May 26, 2026).

[53] Wendt I. EU Competition Law and Liberal Professions: An Uneasy Relationship? Dordrecht: Martinus Nijhoff Publishers (2012).

[54] European Union. Directive 2006/123/EC on services in the internal market. Official Journal of the European Union, L 376 (2006). p. 36–68. Available online at: https://eur-lex.europa.eu/legal-content/EN/TXT/PDF/?uri=CELEX:32006L0123 (Accessed May 26, 2026).

[55] de Waal F. Are We Smart Enough to Know How Smart Animals Are? New York, NY: W. W. Norton and Company (2017).

[56] World Organisation for Animal Health (WOAH). Animal Welfare Mandate. (2002). Available online at: https://www.woah.org/en/who-we-are/structure-and-governance/framework/basic-texts/new-mandates/ (Accessed May 26, 2026).

[57] World Organisation for Animal Health (WOAH). Terrestrial Animal Health Code: Chapter 7.12 – Welfare of working equids. (n.d.). Available online at: https://www.woah.org/fileadmin/Home/eng/Health_standards/tahc/2018/en_chapitre_aw_working_equids.htm (Accessed May 26, 2026).

[58] Belgium. Constitution of Belgium. (n.d.). Available online at: https://www.belgischegrondwet.be/de-belgische-grondwet/huidige-grondwet (Accessed May 26, 2026).

[59] De Briyne N Vidović J Morton D Magalhães-Sant'Ana M. Evolution of the teaching of animal welfare science, ethics and law in European veterinary schools (2012–2019). Animals. (2020) 10:1238. doi: 10.3390/ani10071238 32708281 PMC7401564

[60] European Union. Regulation (EU) 2019/6 of the European Parliament and of the Council of 11 December 2018 on veterinary medicinal products and repealing Directive 2001/82/EC. Official Journal of the European Union, L 4. (2019). p. 43–167. Available online at: http://data.europa.eu/eli/reg/2019/6/oj (Accessed May 26, 2026).

[61] Toma L Stott AW Heffernan C Ringrose S Gunn GJ. Determinants of biosecurity behaviour of British cattle and sheep farmers – a behavioural economics analysis. Prev Vet Med. (2013) 108:321–33. doi: 10.1016/j.prevetmed.2012.11.009 23194894

[62] James AD Rushton J. The economics of foot and mouth disease. Rev Sci Tech. (2002) 21:637–44. doi: 10.20506/rst.21.3.1356 12523703

[63] Shortall O Ruston A Green M Brennan M Wapenaar W Kaler J. Broken biosecurity? Veterinarians' framing of biosecurity on dairy farms in England. Prev Vet Med. (2016) 132:20–31. doi: 10.1016/j.prevetmed.2016.06.001 27664445

[64] Marić M Manghnani V Niemi J Niine T de Briyne N Jansen W. Empowering veterinary herd health management: insights into education, implementation, and regulation across Europe. Vet Sci. (2024) 11:528. doi: 10.3390/vetsci11110528 39591302 PMC11598985

[65] Gerber M Dürr S Bodmer M. Decision-making of Swiss farmers and the role of the veterinarian in reducing antimicrobial use on dairy farms. Front Vet Sci. (2020) 7:565. doi: 10.3389/fvets.2020.00565 33005642 PMC7479233

[66] Instituut voor Recht en Ethiek in de Diergeneeskunde (IRED). Het melden van dierenverwaarlozing en dierenmishandeling door dierenartsen: deontologische en juridische analyse en advies aan de overheid. (2023). Available online at: https://www.ugent.be/di/nl/faculteit/publicaties/adviesmeldenverwaarlozing.pdf/view (Accessed May 26, 2026).

[67] Wood P. Sole proprietorship vs. incorporation. (2019). Available online at: https://www.mdpi.com/2813-9372/2/1/15 (Accessed May 26, 2026).

[68] Federation of Veterinarians of Europe. VETsurvey. (2023). Available online at: https://fve.org/vetsurvey-is-now-available/ (Accessed December 1, 2025).

[69] Competition and Markets Authority. CMA identifies multiple concerns in vets market. (2024). Available online at: https://www.gov.uk/government/news/cma-identifies-multiple-concerns-in-vets-market (Accessed May 26, 2026).

[70] Ministerie van Landbouw Visserij Voedselzekerheid en Natuur. Onderzoek naar de prijsontwikkelingen in de diergeneeskundige zorg: eindrapport. (2024). Available online at: https://open.overheid.nl/documenten/edede1a5-35f1-41c2-83a1-c81e17e10b63/file (Accessed May 26, 2026).

[71] Hogeveen H Jaspers P Mourits M. Regulering van diergeneeskundige zorg in Europa: een vergelijkende analyse. Wageningen University and Research. (2024). Available online at: https://open.overheid.nl/documenten/73ed9854-814d-46ba-9adc-8a4c6c4de650/file (Accessed May 26, 2026).

[72] Autorité de la Concurrence. Avis n° *25-A-12 du 13 octobre 2025 relatif aux conditions de fixation du prix des médicaments vétérinaires et à l'évolution du coût des soins vétérinaires*. (2025). Available online at: https://fve.us10.list-manage.com/track/click?u=43c46b0b512305332e33bbbf4andid=3cf807a7c9ande=fb653a9d5e (Accessed May 26, 2026).

[73] Williams A. AI in veterinary medicine: where to start and what's new? Vet Rec. (2025) 196:219. doi: 10.1002/vetr.5339 40084776

[74] Albrecht R. AI and the Future of the Veterinary Practice, presented at Fetch Coastal. Atlantic City, NJ: dvm360 (2023).

[75] Bashran PS Appleby RB. What's in the box? A toolbox for safe deployment of artificial intelligence in veterinary medicine. J Am Vet Med Assoc. (2024) 262:1090–8. doi: 10.2460/javma.24.01.0027 38599232

[76] Xiao S Dhand NK Wang Z Hu K Thomson PC House JK . Review of applications of deep learning in veterinary diagnostics and animal health. Front Vet Sci. (2025) 12:1511522. doi: 10.3389/fvets.2025.1511522 40144529 PMC11938132

[77] Beauchamp TL Childress JF. Principles of Biomedical Ethics, 5th Edn. New York: Oxford University Press (2001).

[78] Coghlan S Quinn T. Ethics of using artificial intelligence (AI) in veterinary medicine. AI Soc. (2024) 39:2337–48. doi: 10.1007/s00146-023-01686-1

[79] European Association for Quality Assurance in Higher Education. (n.d.). Available online at: https://www.enqa.eu (Accessed May 26, 2026).

[80] Cicero MT. (44 BCE) De Officiis, I, paras. 151 and 155. Available online at: https://droitromain.univ-grenoble-alpes.fr/Auteurs_anciens/deofficiis1_lat.htm (Accessed May 26, 2026).

